# Comparison of stone-free rates following shock wave lithotripsy, percutaneous nephrolithotomy, and retrograde intrarenal surgery for treatment of renal stones: A systematic review and network meta-analysis

**DOI:** 10.1371/journal.pone.0211316

**Published:** 2019-02-21

**Authors:** Doo Yong Chung, Dong Hyuk Kang, Kang Su Cho, Won Sik Jeong, Hae Do Jung, Jong Kyou Kwon, Seon Heui Lee, Joo Yong Lee

**Affiliations:** 1 Department of Urology, Severance Hospital, Urological Science Institute, Yonsei University College of Medicine, Seoul, Korea; 2 Department of Urology, Inha University School of Medicine, Incheon, Korea; 3 Department of Urology, Gangnam Severance Hospital, Urological Science Institute, Yonsei University College of Medicine, Seoul, Korea; 4 Department of Urology, Kwangju Christian Hospital, Gwangju, Korea; 5 Department of Urology, Yongin Severance Hospital, Yonsei University Health System, Yongin, Korea; 6 Department of Urology, Severance Check-Up, Yonsei University Health System, Seoul, Korea; 7 Department of Nursing Science, Gachon University College of Nursing, Incheon, Korea; All India Institute of Medical Sciences, Bhopal, INDIA

## Abstract

**Objectives:**

To perform a systematic review and network meta-analysis comparing stone-free rates following retrograde intrarenal surgery (RIRS), extracorporeal shock wave lithotripsy (SWL), and percutaneous nephrolithotomy (PCNL) treatments of renal stones.

**Materials and methods:**

Clinical trials comparing RIRS, SWL, and PCNL for treatment of renal stones were identified from electronic databases. Stone-free rates for the procedures were compared by qualitative and quantitative syntheses (meta-analyses). Outcome variables are shown as risk ratios (ORs) with 95% credible intervals (CIs).

**Results:**

A total of 35 studies were included in this network meta-analysis of success and stone-free rates following three different treatments of renal stones. Six studies compared PCNL versus SWL, ten studies compared PCNL versus RIRS, fourteen studies compared RIRS versus SWL, and five studies compared PCNL, SWL, and RIRS. The quality scores within subscales were relatively low-risk. Network meta-analyses indicated that stone-free rates of RIRS (OR 0.38; 95% CI 0.22–0.64) and SWL (OR 0.12; 95% CI 0.067–0.19) were lower than that of PCNL. In addition, stone-free rate of SWL was lower than that of RIRS (OR 0.31; 95% CI 0.20–0.47). Stone free rate of PCNL was also superior to RIRS in subgroup analyses including ≥ 2 cm stone (OR 4.680; 95% CI 2.873–8.106), lower pole stone (OR 1.984; 95% CI 1.043–2.849), and randomized studies (OR 2.219; 95% CI 1.348–4.009). In rank-probability test, PCNL was ranked as No. 1 and SWL was ranked as No. 3.

**Conclusions:**

PCNL showed the highest success and stone-free rate in the surgical treatment of renal stones. In contrast, SWL had the lowest success and stone-free rate.

## Introduction

Urinary tract calculi, one of the most common benign urological diseases, is seen in 12% of patients and has a recurrence rate of approximately 50% [1, 2]. Factors that may play an important role in the increase of urinary tract stone disease include increases in diagnosis of metabolic syndrome, lifestyle changes, dehydration, lack of water intake, and low urine volume [3]. Furthermore, recent studies have shown that the worldwide increase of renal colic and renal stones is affected by seasonal changes, particularly the hot season, and that global warming is capable of increasing the incidence of renal stones [4]. In particular, renal uric acid stones show a tendency to increase in hot and dry climates because of the reduction of urine excretion and urine pH [5].

The European Association of Urology (EAU) Urolithiasis Guidelines suggest that the primary treatment of renal stones <2 cm should include extracorporeal shock wave lithotripsy (SWL) and retrograde intrarenal surgery (RIRS) and that the primary treatment for renal stones >2 cm should include percutaneous nephrolithotomy (PCNL) [6]. In cases of 1–2-cm lower pole renal stones, RIRS or PCNL is recommended if there are unfavorable factors in SWL. In comparison with PCNL and RIRS, SWL plays a pivotal role in the treatment of urinary tract stones because it is the only interventional treatment with non-invasive properties [7]. In contrast with SWL, RIRS can perform stone dusting and fragmentation under endoscopic direct vision and has the advantage of being able to directly remove the fragmented stone using a stone basket [8]. PCNL is the standard treatment for large, renal stones (>2 cm) and can also be considered as a treatment option for large stones with resistance to shock waves [9]. Though prospective studies and a meta-analysis of the three treatments along with their advantages and disadvantages have been reported, a network meta-analysis that compares all three treatments at the same time has not yet been reported. Network meta-analysis is a research method that can compare multiple treatments using direct comparison and indirect comparison methods [10–12]. Therefore, we performed a systematic review and a network meta-analysis analysis that compares the success as well as the stone-free rates of SWL, RIRS, and PCNL.

## Materials and methods

### Inclusion criteria

Published clinical studies that were in accordance with the following criteria were included: (i) study design assessed two or three methods, including SWL, PCNL, and RIRS, to treat renal stones; (ii) baseline characteristics of patients from two or three groups were matched, including the total number of subjects and the values of each index; (iii) outcomes of SWL, PCNL, and RIRS were analyzed by stone-free or success rates according to each group; (iv) standard indications for SWL, PCNL, and RIRS to treat renal stones were accepted; (v) endpoint outcome parameters also included complication rate; (vi) the full text of the study was available in English. This report was prepared in compliance with the Preferred Reporting Items for Systematic Reviews and Meta-Analyses (PRISMA) statement (accessible at http://www.prisma-statement.org/) [13]. The protocol for this study is shown in S1 Table.

### Search strategy

A literature search of all publications before 31 June 2016 was performed using EMBASE and PubMed. Additionally, a cross-reference search of eligible articles was performed to identify studies that were not found during the computerized search. The proceedings of appropriate meetings were also searched. Combinations of the following MeSH terms and keywords were used: extracorporeal shock wave lithotripsy, shock wave lithotripsy, percutaneous nephrolithotomy, nephrolithotomy, percutaneous, flexible ureteroscopy, flexible ureterorenoscopy, retrograde intrarenal surgery, renal stone, urolithiasis, rate, and stone-free (S2 Table).

### Data extraction

Two researcher (DYC and DHK) screened all titles and abstracts identified by the search strategy. Two other researchers (HDJ and JKK) independently evaluated the full text of each paper to determine whether it met the inclusion criteria. Disagreements were resolved by discussion until a consensus was reached or by arbitration mediated by another researcher (JYL).

### Quality assessment for studies

When the final group of articles was agreed upon, two researchers independently examined the quality of each article using the Downs and Black checklist. The Downs and Black checklist was developed for the purpose of quality assessment of both randomized and nonrandomized studies of health interventions [14]. The checklist consists of five subscales: reporting, internal validity bias, internal validity confounding, external validity, and power. Because six items in the original list were related to intervention, randomization, and power calculation, and not all of the studies examined were randomized studies, the scores for these six items were counted as zero, as suggested in a previous study [15]. Therefore, the maximum quality score was 31 points. A higher score was considered to be an indicator of a good quality study.

### Heterogeneity tests

Heterogeneity of included studies was examined using the Q statistic and Higgins’ I^2^ statistic [16]. Higgins’ I^2^ measures the percentage of total variation due to heterogeneity rather than chance across studies. Higgins’ I^2^ was calculated as follows:

$$I^{2}=\frac{Q-\mathrm{df}}{Q}\times -\mathrm{df},$$

in which “Q” is Cochran's heterogeneity statistic and “df” is the degrees of freedom.

An I^2^ with I degrees of freedom represents substantial heterogeneity [17]. For the Q statistic, heterogeneity was deemed to be significant for p<0.10 [18]. If there was evidence of heterogeneity, the data were analyzed using a random-effects model. Studies in which positive results had been confirmed were assessed with a pooled specificity using 95% CIs. In addition, L’Abbe plot and Galbraith’s radial plot were created to evaluate heterogeneity [19, 20].

### Ethics statement

The study was exempt from requiring the participants’ written informed consent because this is systematic review and network meta-analysis. The approval of the Institutional Review Board was also exempted.

### Statistical analysis

Outcome variables measured at specific time points were compared in terms of odds ratios (OR) or mean differences with 95% CIs using a network meta-analysis. Analyses were based on non-informative priors for effect sizes and precision. Convergence and lack of auto-correlation were confirmed after four chains and a 50,000-simulation burn-in phase. Finally, direct probability statements were derived from an additional 100,000-simulation phase. The probability that each group had the lowest rate of clinical events was assessed by Bayesian Markov Chain Monte Carlo modeling. Sensitivity analyses were performed by repeating the main computations with a fixed-effect method. Model fit was appraised by computing and comparing estimates for deviance and deviance information criterion. All statistical analyses were performed with R (R version 3.5.1, R Foundation for Statistical Computing, Vienna, Austria; http://www.r-project.org) and the associated meta, netmeta, pcnetmeta, and gemtc packages for pairwise and network meta-analyses.

## Results

### Eligible studies

**Fig 1:**
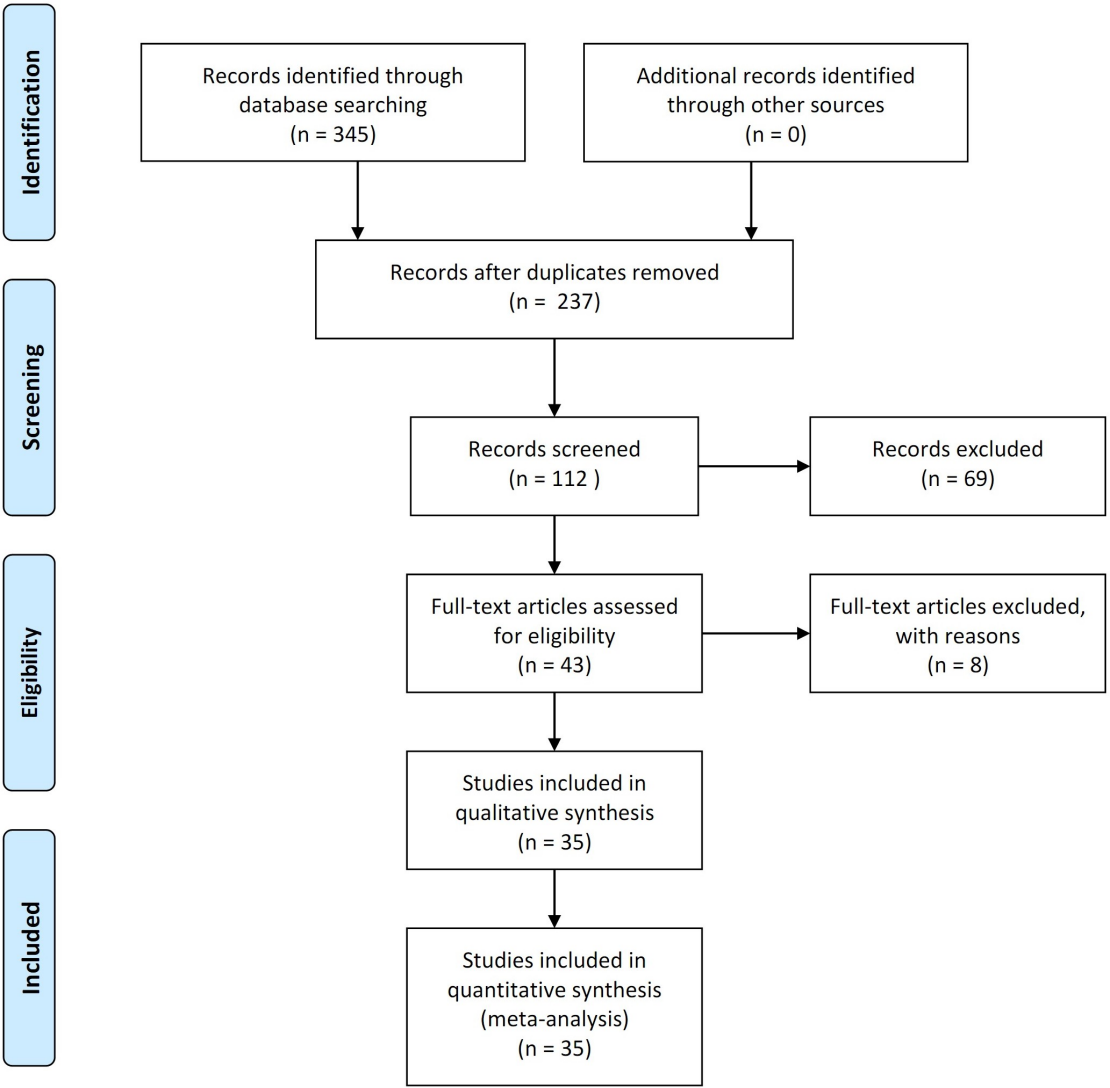
Flow diagram of evidence acquisition. Thirteen studies were ultimately included in the qualitative and quantitative review that used pairwise and network meta-analyses.

The database search retrieved 35 articles covering 237 studies for potential inclusion in meta-analysis. Eight articles were excluded according to the inclusion/exclusion criteria; three had no data on stone-free rate, three were reviews, and two reported case series. The remaining 35 articles were included in the qualitative and quantitative syntheses using pairwise and network meta-analyses (Fig 1).

**Fig 2:**
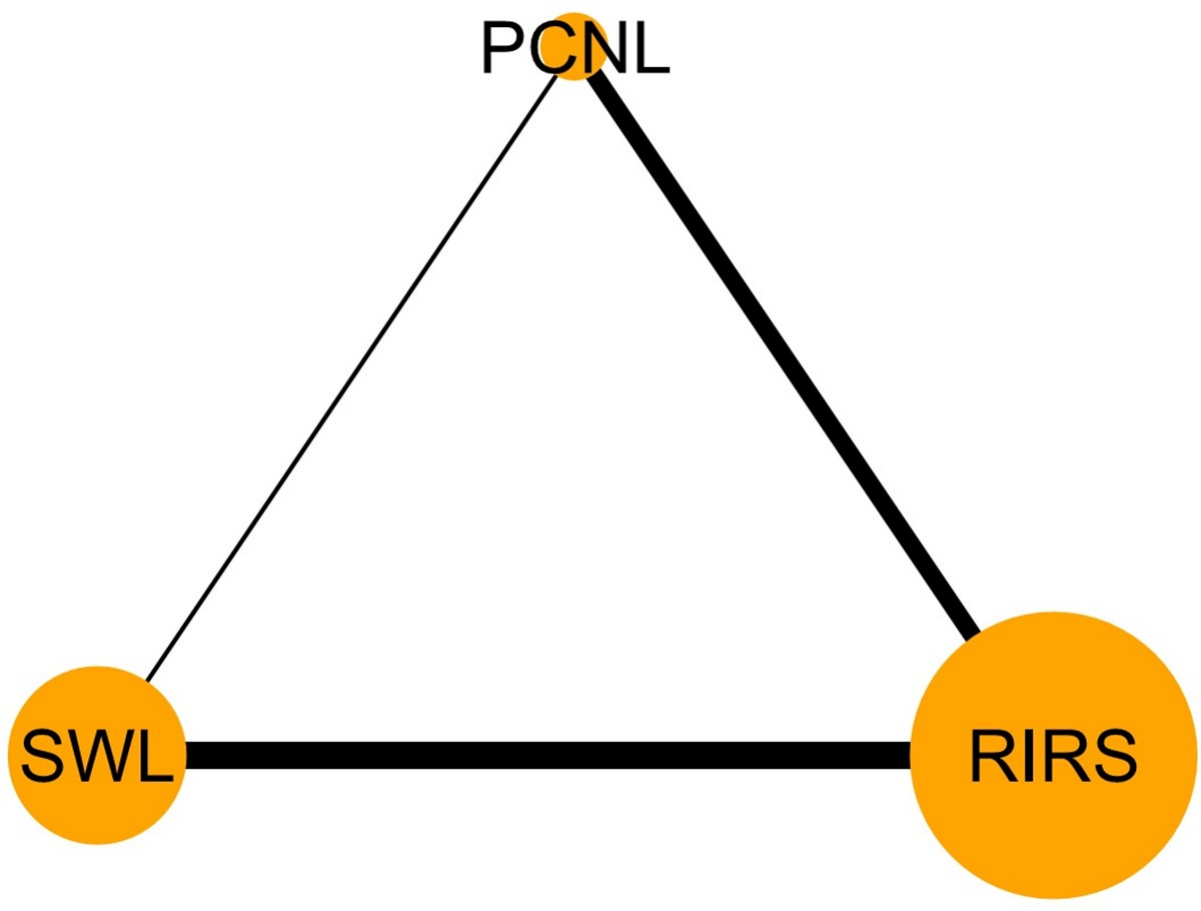
Network plots for included studies. Six studies compared PCNL versus SWL. Six studies reported outcomes between PCNL and RIRS. Eight studies compared outcomes between RIRS and SWL. Four studies demonstrated the comparison for PCNL, SWL, and RIRS.

Data corresponding to confounding factors derived from each study are summarized in Table 1. Six studies compared PCNL and SWL [21–26]. Ten trials reported outcomes between PCNL and RIRS [27–36]. Fourteen studies compared outcomes between RIRS and SWL [37–50]. Five articles compared PCNL, SWL, and RIRS [51–55] (Fig 2). Stone-free rates of enrolled studies are summarized in Table 1.

**Table 1:** Enrolled studies for current meta-analysis.

Category	Study	Year	Methods	Study Design	Inclusion Criteria	No. of Patients	Follow-up	Definition of Stone-free	Stone-free Patients (No.)	Stone-free Rate (%)	Complication (No.)		Quality Assessment
											Clavien I-II	Clavien III-IV	
PCNL vs. SWL	Netto et al. [21]	1991	PCNL	Retrospective	≤ 3 cm, single or multiple stones	23	3 months	Complete removal	22	95.7	3	0	13
			SWL			24	3 months		19	79.2	1	0	
	Havel et al. [22]	1998	PCNL	Retrospective	Solitary lower pole caliceal calculi	73	1 day	Not stated	53	72.6	51	5	15
			SWL			587	3 months		335	57.1	88	5	
	Albala et al. [23]	2001	PCNL	Randomized controlled	Symptomatic lower pole, ≤ 3 cm	55	3 months	Not stated	52	94.5	12.0	2	14
			SWL			52	3 months		19	36.5	6.0	1	
	Preminger et al. [24]	2006	PCNL	Randomized controlled	Solitary lower pole stone, ≤ 3cm	47	3 months	Not stated	45	95.7	Not stated		14
			SWL			54	3 months		19	35.2			
	Yuruk et al. [25]	2010	PCNL	Randomized controlled	Asymptomatic lower caliceal, ≤ 2 cm	31	3 months	Not stated	30	96.8	2.0	0	13
			SWL			31	3 months		17	54.8	2.0	0	
	Hassan et al. [26]	2015	PCNL	Retrospective	2 to 3 cm, renal pelvis stone	170	Not stated	Not stated	162	95.3	13.0	0	17
			SWL			167	Not stated		115	68.9	4.0	0	
PCNL vs. RIRS	Hyams et al. [27]	2009	PCNL	Retrospective	2–3 cm, renal stone	20	3 months	< 4 mm	20	100.0	Not stated		13
			RIRS			19	3 months		18	94.7			
	Akman et al. [28]	2011	PCNL	Retrospective	2–4 cm, renal stone	34	3 months	Not stated	33	97.1	4.0	1	14
			RIRS			34	3 months		32	94.1	3.0	1	
	Bozkurt et al. [29]	2011	PCNL	Retrospective	1.5–2 cm, renal stone	42	2 procedures	Not stated	41	97.6	7.0	0	13
			RIRS			37	2 procedures		35	94.6	4.0	0	
	Bryniarski et al. [30]	2012	PCNL	Randomized controlled	Renal pelvis stone, ≥ 2 cm	32	3 weeks	Not stated	30	93.8	Not stated		14
			RIRS			32	3 weeks		24	75.0			
	Jung et al. [31]	2015	PCNL	Retrospective	1.5–3 cm, lower pole stone	44	1 month	< 3 mm	37	84.1	5.0	2	16
			RIRS			44	1 month		41	93.2	1.0	1	
	Karakoyunlu et al. [32]	2015	PCNL	Randomized controlled	Renal pelvis stone, > 2 cm	30	Final procedures	Complete removal	26	86.7	15.0	0	17
			RIRS			30	Final procedures		20	66.7	19.0	0	
	Koyuncu et al. [33]	2015	PCNL	Retrospective	Lower pole stones, ≥ 2 cm	77	Final procedures	Complete removal	74	96.1	4.0	1	14
			RIRS			32	Final procedures		29	90.6	3.0	0	
	Bas et al. [34]	2015	PCNL	Retrospective	Symptomatic stone-bearing calyceal diverticula	29	3 months	Less than 3 mm	24	82.8	3.0	3	13
			RIRS			25	3 months		19	76.0	4.0	1	
	Zengin et al. [35]	2015	PCNL	Retrospective	Kidney stones, ≥ 2–3 cm	74	1 month	Less than 2 mm	71	95.9	8.0	2	14
			RIRS			80	1 month		65	81.3	7.0	0	
	Ozayar et al. [36]	2016	PCNL	Prospective	Lower pole stone, ≤ 2 cm	30	Not stated	Not stated	28	93.3	Not stated		13
			RIRS			26			23	88.5			
SWL vs. RIRS	Pearle et al. [37]	2008	SWL	Randomized controlled	Isolated lower pole stone, < 1 cm	26	3 months	Complete removal	9	34.6	6.0	1	12
			RIRS			32	3 months		16	50.0	6.0	1	
	Koo et al. [38]	2011	SWL	Retrospective	Lower pole renal calculi, ≤ 2 cm	51	Final procedures	Complete removal	30	58.8	2.0	2	15
			RIRS			37	Final procedures		24	64.9	1.0	3	
	El-Nahas et al. [39]	2012	SWL	Retrospective	Lower pole stones, 1 to 2 cm	62	3 months	Complete removal	42	67.7	2.0	1	16
			RIRS			37	3 months		32	86.5	4.0	1	
	Salem et al. [40]	2013	SWL	Randomized controlled	Renal stone, ≤ 2 cm	30	3 months	< 3 mm	17	59.7	7.0	0	14
			RIRS			30	3 months		29	96.7	5.0	0	
	Sener et al. [41]	2014	SWL	Randomized controlled	Lower pole stones, < 1 cm	70	3 months	Not stated	64	91.4	3.0	1	16
			RIRS			70	3 months		70	100.0	3.0	0	
	Singh et al. [42]	2014	SWL	Randomized controlled	Inferior calyceal stones, 1 to 2 cm	35	1 month	Not stated	17	48.3	15.0	2	14
			RIRS			35	1 month		29	82.9	10.0	1	
	Burr et al. [43]	2015	SWL	Retrospective	Lower pole stones	93	6–12 weeks	Less than 3 mm	23	24.7	3.0	0	14
			RIRS			68	6–12 weeks		63	92.6	4.0	0	
	Kumar et al. [44]	2015	SWL	Randomized controlled	Lower calyceal calculi, ≤ 2 cm	90	3 months	Radiologic absence of stone	74	82.2	6.0	0	15
			RIRS			90	3 months		78	86.7	10.0	0	
	Sener et al. [45]	2015	SWL	Randomized controlled	Asymptomatic lower pole, < 1 cm	50	3 months	Not stated	45	90.0	4.0	2	15
			RIRS			50	3 months		46	92.0	8.0	6	
	Tauber et al. [46]	2015	SWL	Retrospective	Renal stone, ≤ 1.5 cm	165	6–12 weeks	Radiologic absence of stone	71	43.0	9.0	10	14
			RIRS			161	6–12 weeks		134	83.2	6.0	11	
	Vilches et al. [47]	2015	SWL	RCT	Lower pole stone, ≤ 1.5 cm	31	2 months	Less than 3 mm	15	48.4	19.0	0	15
			RIRS			24	2 months		17	70.8	17.0	0	
	Yuruk et al. [48]	2015	SWL	Retrospective	Renal stone in solitary kidney patients	30	3 months	Radiologic absence of stone	22	73.3	4.0	11	14
			RIRS			18	3 months		12	66.7	2.0	5	
	Gokce et al. [49]	2016	SWL	Retrospective	horsehoe kidney (16.8±4.4 mm) (lower 12, pelvis upper 32)	44	6 weeks	Less than 3 mm	21	47.7	8.0	0	14
			RIRS			23	6 weeks		17	73.9	7.0	0	
	Javanmard et al. [50]	2016	SWL	RCT	Renal stone, 0.6 cm-2 cm	60	3 months	Radiologic absence of stone	45	75.0	13.0	5	16
			RIRS			60	3 months		52	86.7	5.0	0	
PCNL vs. SWL vs. RIRS	Aboutaleb et al. [51]	2012	PCNL	Retrospective	Lower calyceal stone, 1–2 cm	19	2 days	< 3 mm considered insignificant	17	89.5	6.0	0	15
			SWL			24	Not stated		15	62.5	10.0	0	
			RIRS			13	2 days		11	84.6	6.0	0	
	Resorlu et al. [52]	2013	PCNL	Retrospective	Radiolucent renal calculi, 1–2 cm	140	1 procedure	Not stated	128	91.4	28.0	3	17
			SWL			251	Final session		167	66.5	19.0	0	
			RIRS			46	1 procedure		40	87.0	5.0	0	
	Ozturk et al. [53]	2013	PCNL	Retrospective	Renal stone, 1.5–2 cm	144	Not stated	Less than 3 mm	135	93.8.	14.0	5	16
			SWL			221	4 months		168	76.0	5.0	2	
			RIRS			38	Not stated		28	73.7	1.0	1	
	Bas et al. [54]	2014	PCNL	Retrospective	Renal pelvis stone, ≥ 2 cm	50	1 month	Not stated	49	98.0	4.0	2	17
			SWL			52	Mean 2.6 sessions		45	86.5	3.0	1	
			RIRS			47	1 month		43	91.5	2.0	1	
	Kumar et al. [55]	2015	PCNL	RCT	Radiolucent lower pole renal calculi, 1–2 cm	41	3 months	Less than 4 mm	39	95.1	10.0	0	18
			SWL			42	3 months		31	73.8	3.0	0	
			RIRS			43	3 months		37	86.0	4.0	0	

PCNL, percutaneous nephrolithotomy; SWL, shock wave lithotripsy; RIRS, retrograde intrarenal surgery

PCNL (1,205 cases), SWL (2,342 cases), RIRS (1,281 cases)

### Quality assessment

The results of quality assessment based on the Downs and Black checklist are shown in Table 1. The median of the total quality scores was 14.8. Overall, the quality scores within subscales were relatively low. In most studies, external validity was not satisfactory for both significant and insignificant groups.

### Heterogeneity and inconsistency assessment and publication bias

**Fig 3:**
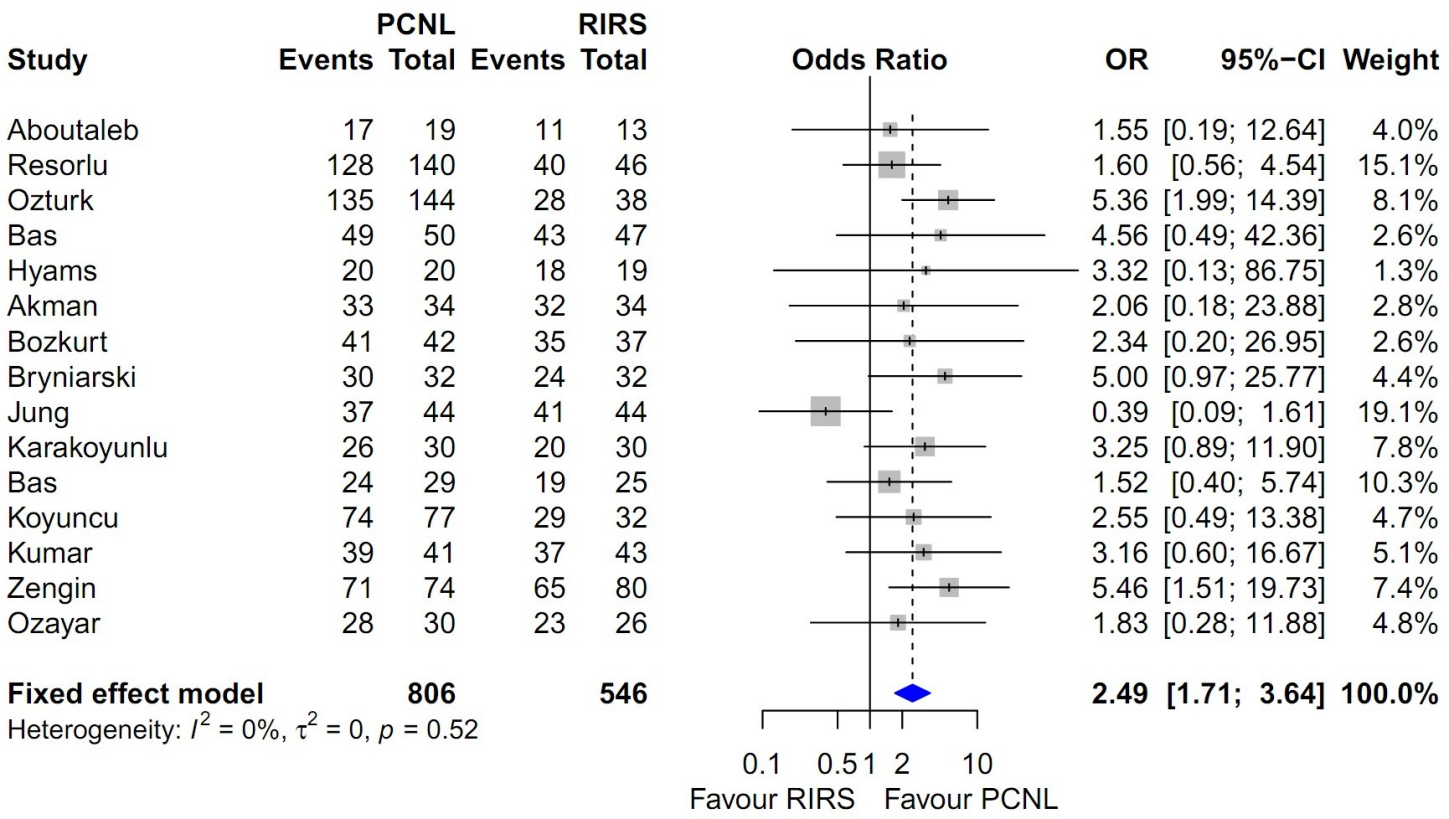
Pairwise meta-analysis of success rate in PCNL and RIRS. Pooled data assessment of stone-free rate between PCNL and RIRS showing a significantly higher stone-free rate with PCNL (OR 2.31; 95% CI 1.45–3.67; P<0.001).

**Fig 4:**
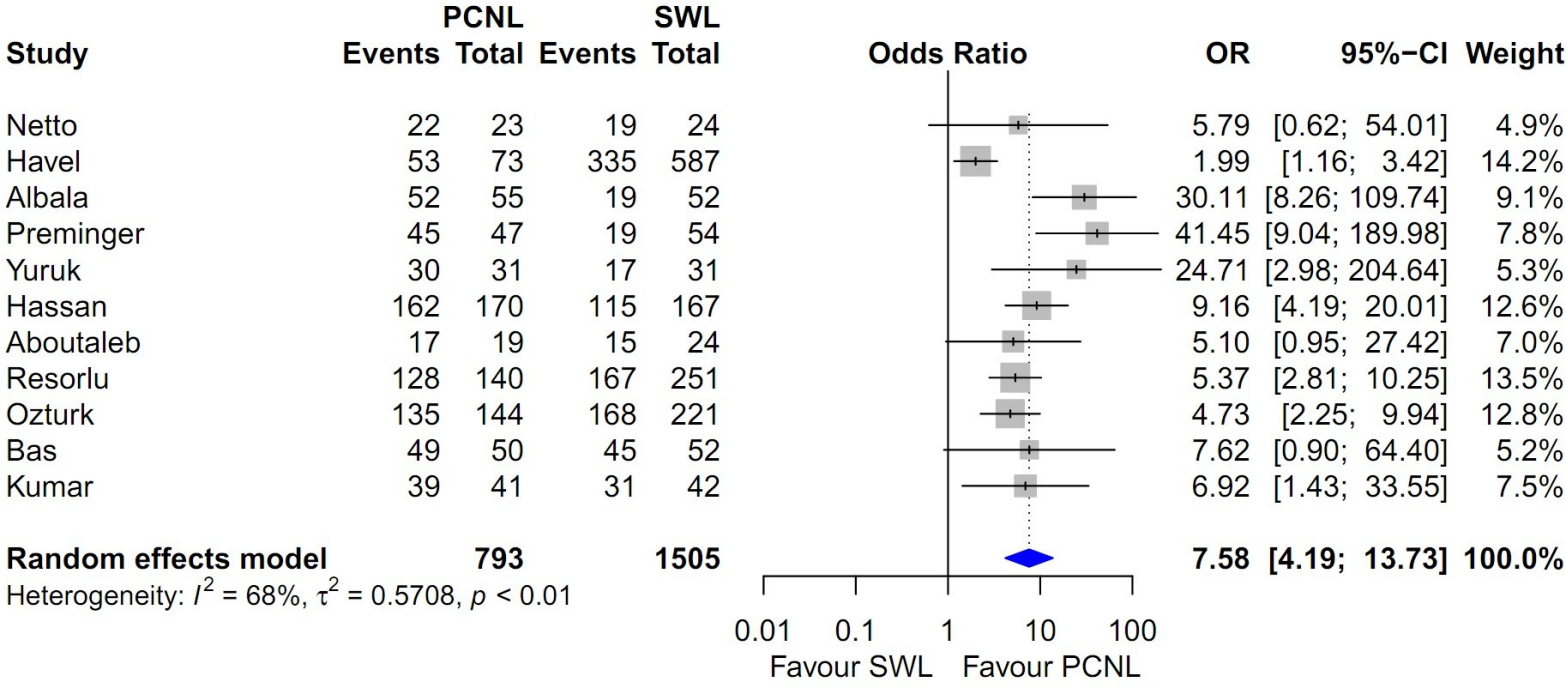
Pairwise meta-analysis of success rate in PCNL and SWL. Results show that the stone-free rate of PCNL was superior to SWL (OR 7.71; 95% CI 4.08–14.57; P<0.001).

**Fig 5:**
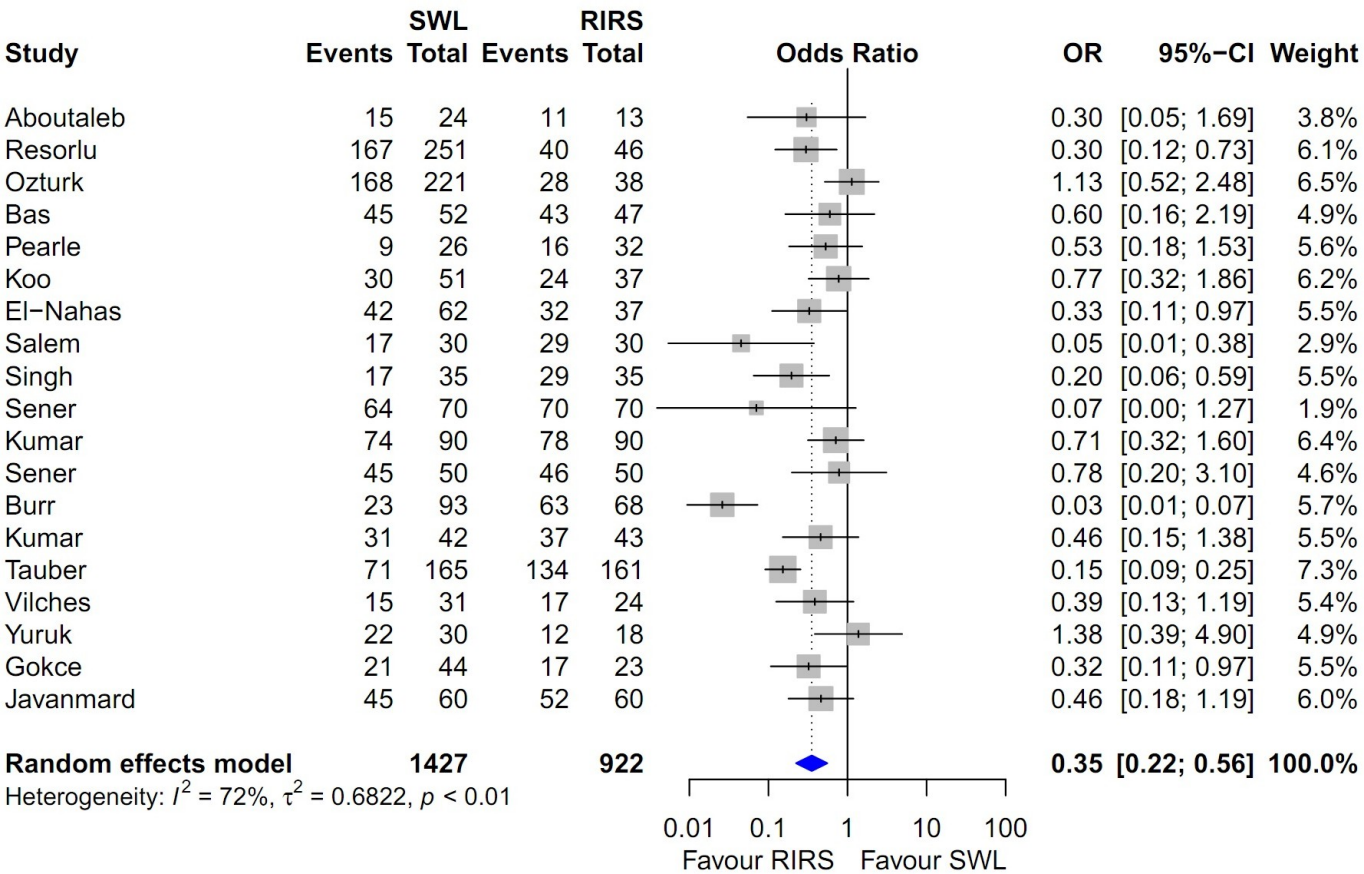
Pairwise meta-analysis of success rate in SWL and RIRS. Results show that the stone-free rate of SWL was lower than RIRS (OR 60.46; 95% CI 0.30–0.71; P<0.001).

**Fig 6:**
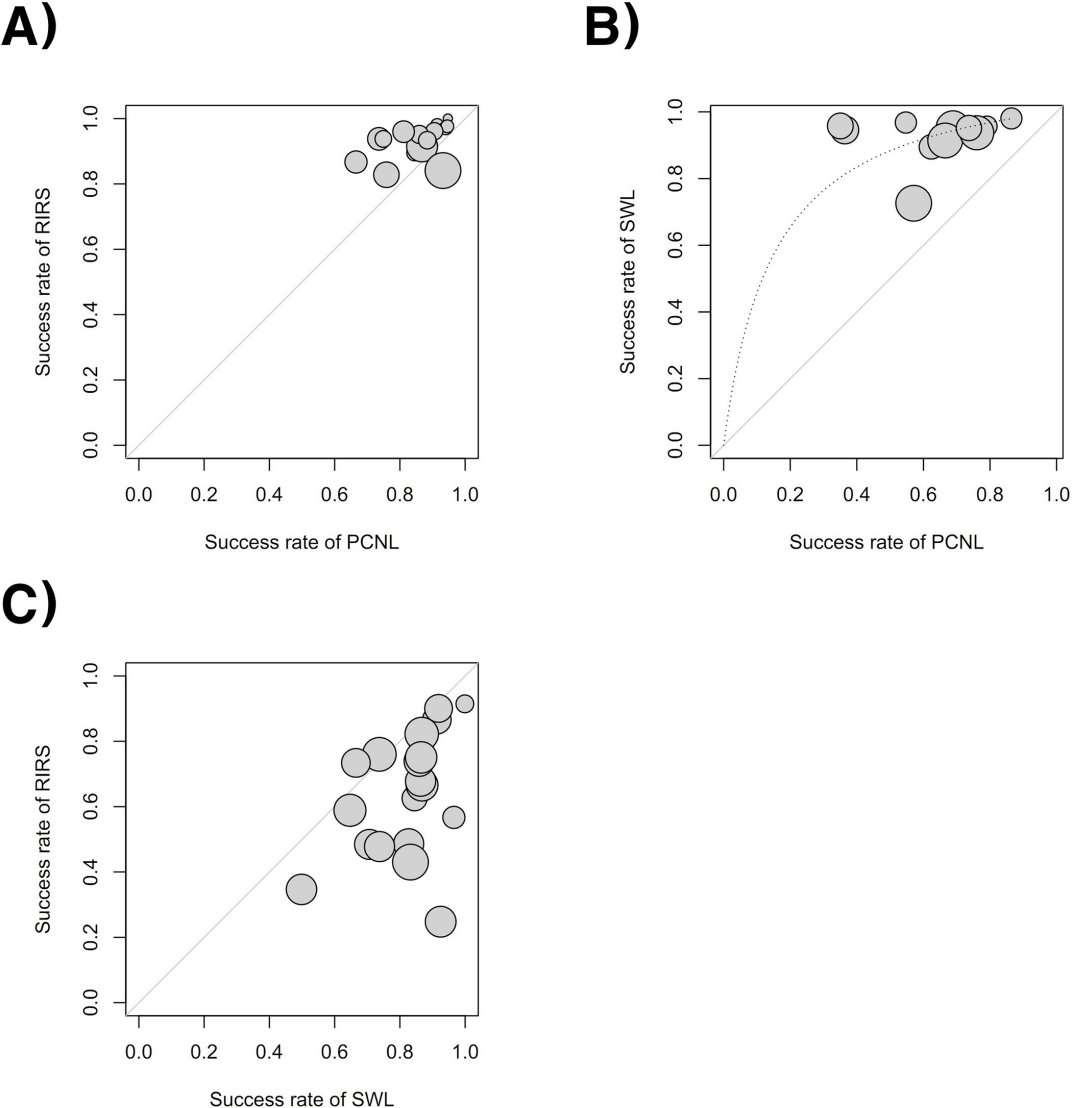
L’Abbe plots of success rate between RIRS and PCNL (A), SWL and PCNL (B) and RIRS and SWL (C). Little heterogeneity was noted in L’Abbe plots.

**Fig 7:**
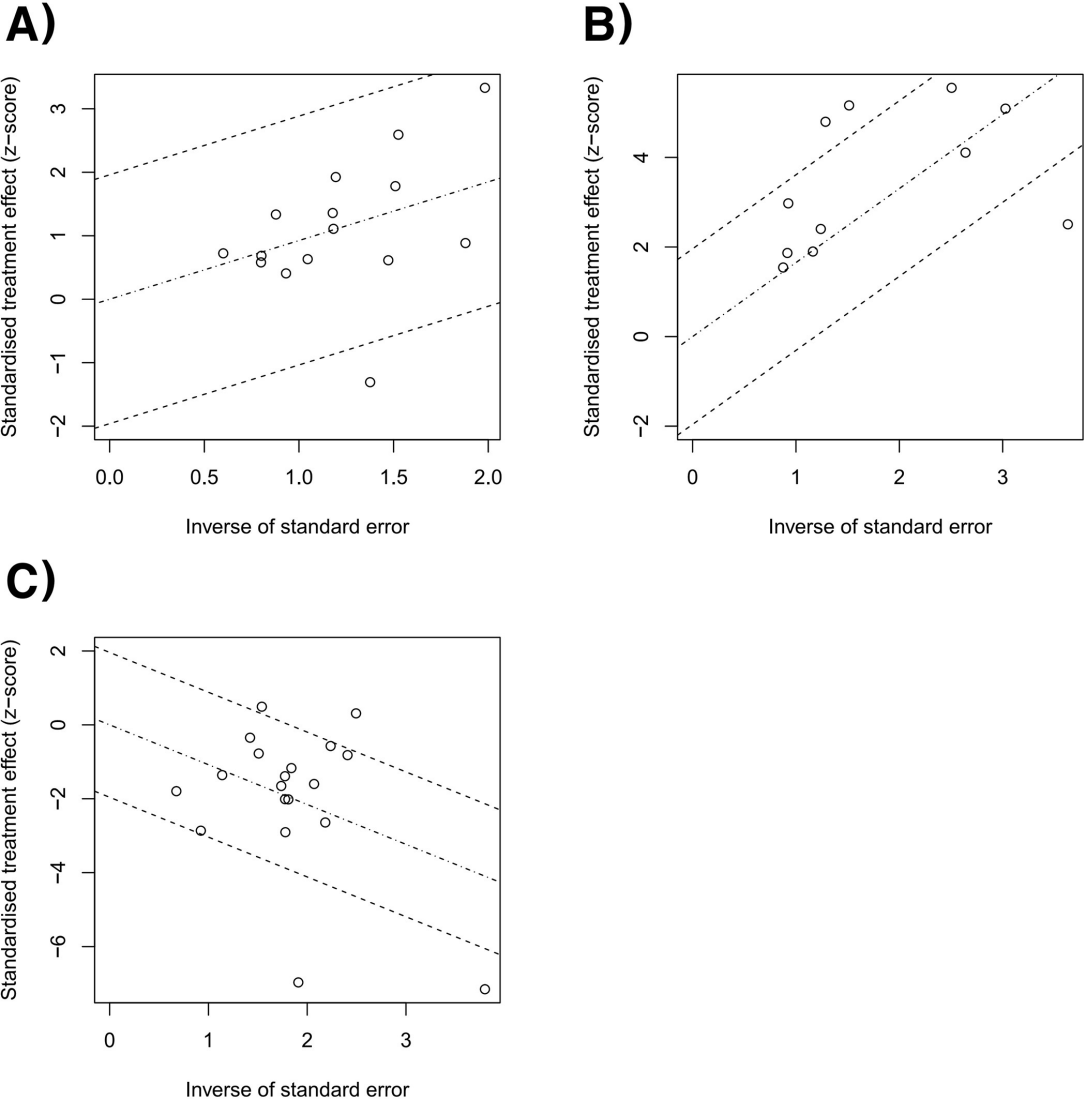
Radial plots of success rate between RIRS and PCNL (A), SWL and PCNL (B), and RIRS and SWL (C). Little heterogeneity was noted in radial plots.

Forest plots of the pairwise meta-analysis of SWL, PCNL, and RIRS are shown in Figs 3, 4 and 5, respectively. There was no heterogeneity between PCNL and RIRS; however, there was heterogeneity between PCNL and SWL and between SWL and RIRS in each study. Thus, random-effect models were applied using the Mantel–Haenszel method for PCNL and SWL analysis and SWL and RIRS comparison (Figs 4 and 5). After selection of effect models, little heterogeneity was noted in L’Abbe plots and radial plots (Figs 6 and 7).

**Fig 8:**
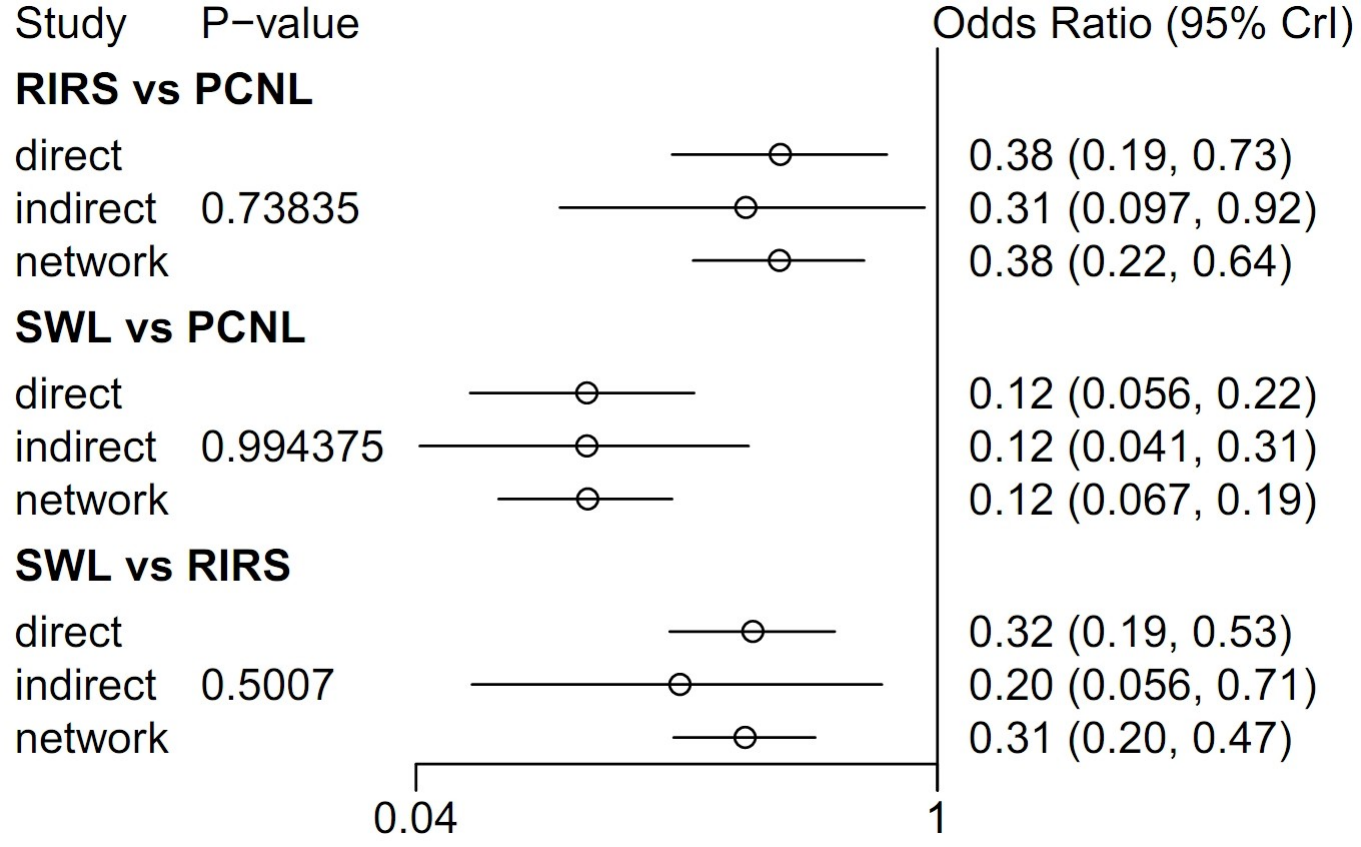
Network meta-analysis for success rate of RIRS, PCNL, SWL, and node-splitting analyses of inconsistency. In node-splitting analysis, no inconsistency was demonstrated in direct, indirect, or network comparison.

**Fig 9:**
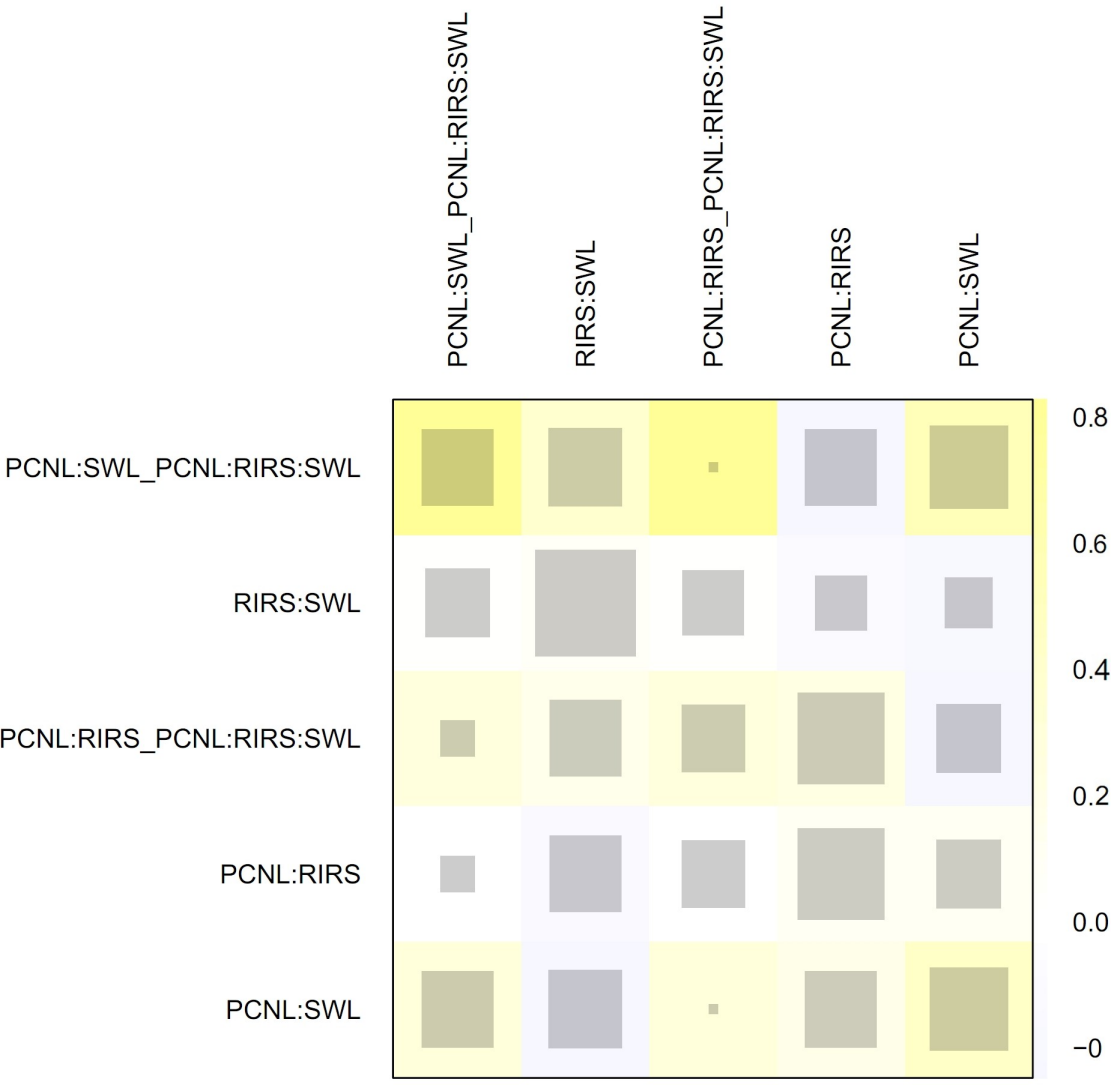
Net-heat plot for inconsistency. Net-heat plot showing that there is little inconsistency in whole network analysis of PCNL, SWL, and RIRS.

In node-splitting analysis, no inconsistency was demonstrated in direct, indirect, or network comparison (Fig 8). A net-heat plot showed that there was also little inconsistency in the whole network (Fig 9).

**Fig 10:**
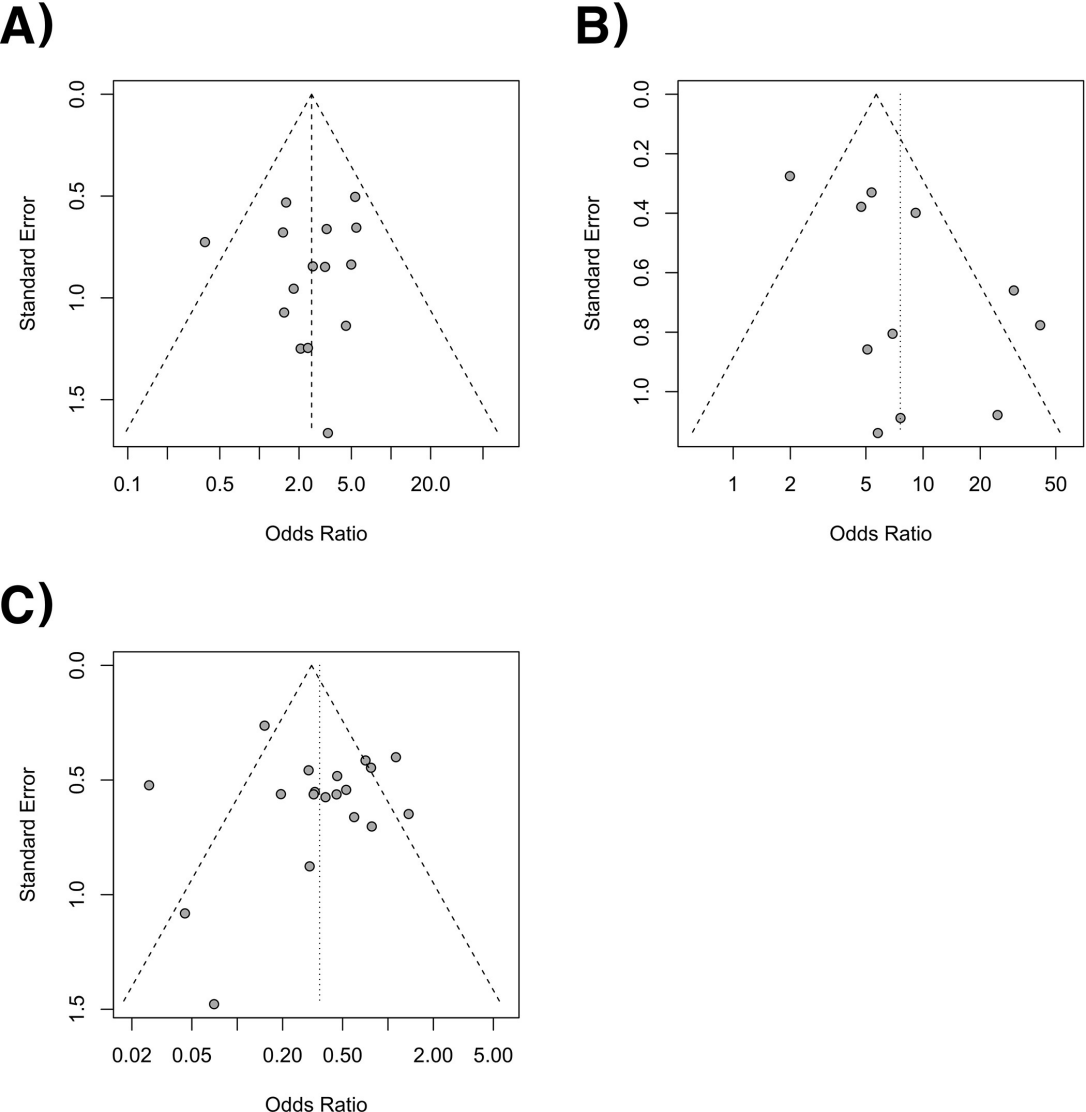
Funnel plots of success rate between RIRS and PCNL (A), SWL and PCNL (B), and RIRS and SWL (C). There were some publication bias in funnel plots.

The Begg and Mazumdar rank correlation tests for each analysis showed no evidence of publication bias in the present meta-analysis between PCNL and SWL (P = 0.697). However, Egger’s regression intercept tests revealed a slight publication bias (P = 0.041). According to a rank correlation test (P = 0.520) and regression tests (P = 0.771), there was no publication bias in PCNL and RIRS. Also, no publication bias was shown for SWL versus RIRS in the rank correlation test (P = 0.421) and regression test (P = 0.855). However, there was little publication bias from funnel plots in each comparison (Fig 10).

### Pairwise meta-analysis of SWL, PCNL, and RIRS for stone-free rate

Pooled data that were used to compare the stone-free rate between PCNL and RIRS showed a significantly higher stone-free rate with PCNL (OR 2.493; 95% CI 1.708–3.637; P<0.001; Fig 3). The stone-free rate of PCNL was superior to that of SWL (OR 7.583; 95% CI 4.188–13.731; P<0.001; Fig 4). The stone-free rate of SWL was lower than that RIRS (OR 0.352; 95% CI 0.223–0.557; P<0.001; Fig 5).

### Network meta-analysis of SWL, PCNL, and RIRS for stone-free rate

**Fig 11:**
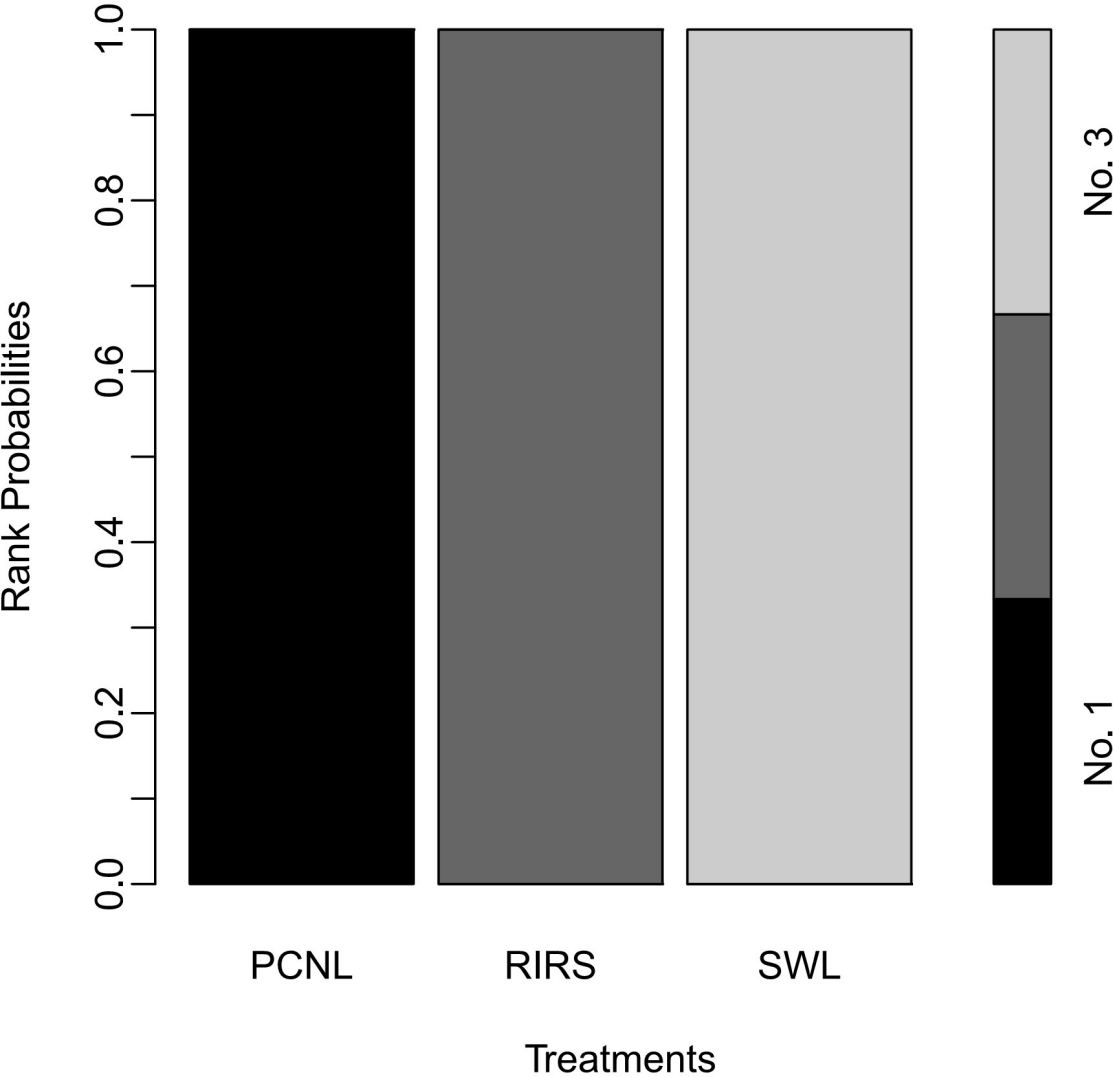
Rank-probability test of network meta-analyses. In the rank-probability test, PCNL was ranked as No. 1 and SWL was ranked as No. 3.

In network meta-analyses, the stone-free rate of RIRS was lower than that of PCNL (OR 0.38; 95% CI 0.22–0.64), the stone-free rate of SWL was lower than that of PCNL (0.12; 95% CI 0.067–0.19), and the stone-free rate of SWL was lower than that of RIRS (OR 0.31; 95% CI 0.20–0.47) (Fig 9). In the rank-probability test, PCNL was ranked as No. 1 and SWL was ranked as No. 3 (Fig 11). The P-score test using a frequentist method to rank treatments in the network demonstrated PCNL (P-score 1.0) was superior to RIRS (P-score 0.5) and SWL (P-score 0) in stone-free rate [56].

### Subgroup analyses using stone size, location of renal stone, and study design

In ≥ 2 cm stones, seven studies were included. There was a single study that compared PCNL to SWL, and there were six studies that demonstrated the comparison between PCNL and RIRS. In this subgroup analysis, PCNL can be superior to RIRS (OR 4.680; 95% CI 2.873–8.106) and SWL (OR 9.732; 95% CI 5.675–28.060), and RIRS can be superior to SWL (OR 2.47; 95% CI 1.076–4.614). In subgroup analysis for lower pole stones, 19 studies were enrolled. The success rate of PCNL can be higher compared to RIRS (OR 1.984; 95% CI 1.043–2.849) and SWL (OR 6.687 95% CI 4.204–10.450). In RCTs, PCNL can be superior to RIRS (OR 2.219; 95% CI 1.348–4.009) and SWL (OR 5.605; 95% CI 3.129–11.250), and RIRS can also be superior to SWL (OR 2.407; 95% CI 1868–3.773) in success rate (Table 2).

**Table 2:** Subgroup network meta-analysis for ≥ 2 cm stone, lower pole stones and RCTs. PCNL, percutaneous nephrolithotomy; SWL, shock wave lithotripsy; RIRS, retrograde intrarenal surgery.

≥ 2 cm		PCNL	RIRS	SWL
	PCNL		4.680 (2.873‒8.106)	9.732 (5.675‒28.060)
	RIRS	0.214 (0.123‒0.348)		2.479 (1.076‒4.614)
	SWL	0.103 (0.036‒0.176)	0.403 (0.217‒0.930)	
Lower pole		PCNL	RIRS	SWL
	PCNL		1.984 (1.043‒2.849)	6.687 (4.204‒10.450)
	RIRS	0.504 (0.351‒0.961)		3.564 (2.398‒5.509)
	SWL	0.150 (0.096‒0.238)	0.281 (0.182‒0.417)	
RCTs		PCNL	RIRS	SWL
	PCNL		2.219 (1.348‒4.009)	5.605 (3.129‒11.250)
	RIRS	0.451 (0.249‒0.742)		2.407 (1.868‒3.773)
	SWL	0.178 (0.089‒0.320)	0.416 (0.265‒0.536)	

### Complication Rate according to Clavien-Dindo classification

From 31 studies, rates of complication in SWL, PCNL, and RIRS were 12.5%, 20.2%, and 15.0%, respectvely. The rate of major complication in total complication cases were 15.4% in SWL, 13.8% in PCNL, and 18.3% in RIRS (Table 3).

**Table 3:** Complication rates from studies according to Clavien-Dindo classification.

Methods		Complication					
		Total		Clavien Grades I-II (Minor)		Clavien Grades III-IV (Major)	
No. of patients		N	%	N	%	N	%
SWL	2,288	287	12.5	243	84.7	44	15.3
PCNL	1,076	217	20.2	187	86.2	30	13.8
RIRS	1,204	180	15.0	147	81.7	33	18.3

## Discussion

The use of minimally invasive techniques like SWL, PCNL, and RIRS, has developed dramatically despite the continued high incidence and recurrence of urinary tract stone disease. [57]. The minimally invasive techniques for treatment of renal stones, have continuously improved over the last 30 years, and new procedures are being introduced as a result of the combination of instruments and technology that is now taking place. Since Fernstrom and Johansson introduced PCNL as the surgical treatment for patients with large and complex renal calculi for the first time in 1976 [58], PCNL has been considered as the standard surgery for the treatment of renal stones >2 cm [9]. The procedure was developed in the sequential order of tubeless PCNL, supine PCNL, and mini-PCNL [59–61]. Further changes in the PCNL procedure led to the recent development of endoscopic combined intrarenal surgery (ECIRS) [62]. The first experience of SWL was reported in 1984, when Chaussy and his colleagues performed SWL on 852 patients [63]. Until recently, the advancement of patient selection, shock wave delivery, and the new lithotripter design were the reasons why SWL is was still the primary treatment for non-lower pole renal stones <2 cm [7]. RIRS has achieved rapid development since the 1990’s when the holmium:yttrium aluminum garnet (YAG) laser system was introduced [64]. The development of the recently introduced small-aperture digital video scope (Flex-Xc; Karl Storz Endoskope, Tuttlingen, Germany, URF-V2; Olympus Corp, Tokyo, Japan) and the single-use video scope (LithoVue; Boston Scientific, Marlborough, MA, USA) has led to the popularization of RIRS by improving both the image quality as well as durability [65, 66].

In most cases of non-symptomatic kidney stones, observation is sufficient. However, treatment is recommended in cases in which stones are continuously increasing in size, there is a high risk of additional stone formation, there is obstruction due to the stones, infection, pain, or hematuria, or stones are >1.5 cm. Treatment is also recommended if it is desired with regard to the patient’s social situation [67]. As mentioned earlier, the EAU guideline suggests SWL and RIRS for the primary treatment of renal stones <2 cm, and PCNL for the primary treatment for stones >2 cm. In general, PCNL is more invasive than RIRS and SWL and has relatively large complications related to hemorrhaging. Though the procedure of SWL is relatively safe, there is a possibility of repeated treatment. RIRS is also expanding in use due to the gradual development of related systems, but there can be technical difficulties and surgical complications may occur. Hence, there are advantages and disadvantages for each interventional treatment, and it is extremely important to find and perform the best treatment for the individual patient with the renal stones.

Perhaps stone-free rate is one of the first things to consider when choosing among treatments that have their own advantages and disadvantages. This report is the first of a network meta-analysis on the success or stone-free rates of SWL, PCNL, and RIRS. A pairwise meta-analysis comparing each method has already been reported several times. In the pairwise meta-analysis of PCNL and RIRS reported in 2015, the complication rate (OR 1.61; 95% CI 1.11–2.35), hemoglobin drop (MD 0.87; 95% CI 0.51–1.22), and the hospital stay (MD 1.28; 95% CI 0.79–1.77) of RIRS showed better results than PCNL [68]. However, the stone-free rate of PCNL was higher than that of RIRS (OR 2.19; 95% CI 1.53–3.13, P<0.001). In our study, the pairwise meta-analysis of PCNL and RIRS showed better results of PCNL in terms of the stone-free rate (OR 2.31; 95% CI 1.45–3.67). Either in the network meta-analysis, RIRS showed a lower stone-free rate than PCNL (OR 0.36; 95% CI 0.19–0.68). In another study, Zhang and colleagues performed pairwise meta-analyses of SWL, PCNL, and RIRS for the lower pole renal stone, and found that PCNL shows a higher stone-free rate than SWL and RIRS, and there is no difference in the stone-free rates of SWL and RIRS (OR 1.97; 95% CI 0.98–3.95) [69]. Our results also show PCNL had the best stone-free rate, but the results for SWL and RIRS differ between our study and that of Zhang et al. These authors argue that residual fragments should be considered more seriously for the lower pole stone than for other locations because gravity plays a crucial role in the clearance of the residual stone fragments. In particular, they predict that the increase in laser dusting without stone extraction in the mini-PCNL and RIRS treatments will play a role in lowering the stone-free rate to values similar to that for the fragments clearance using SWL, and that this prediction explains why the stone-free rate does not differ between SWL and RIRS treatments in their study. Donaldson et al reported meta-analysis on clinical effectiveness of SWL, RIRS and PCNL for lower pole stone [70]. They concluded that PCNL and RIRS were superior to SWL in clearing the stones within 3 months. In their study, they used pair-wise meta-analysis for the outcomes in patients with only lower pole stone. We also performed subgroup analyses with lower pole stone data using Bayesian network meta-analysis and the results of our study also demonstrated similarities to those by Donaldson et al., but we reaffirmed the superiority of PCNL and RIRS using network meta-analysis. In EAU guidelines, in lower pole stone, PCNL and RIRS should be recommended as the first-line treatment [6]. In our analysis, the reason why RIRS showed a higher stone-free rate than SWL was because our research included all renal stones regardless of their location, whereas the analysis performed by Zhang and colleagues included only lower pole renal stones. Furthermore, our results may differ from those of their research because a higher number of studies were included in our meta-analysis. The technical development of RIRS can be another reason for the differing results. A recent survey of 414 surgeons indicates that the dusting technique using high-power holmium laser is popular and that this technique is judged to be a help in improving the stone-free rate of RIRS [71]. The lower pole stone has been reported to be used in 55.8% of cases of translocation using the stone basket. In the case of RIRS and even focusing on the lower pole stones, stones <2 cm may increase the stone-free rate through translocation [72].

There was no difference in the stone-free rate (RR 0.95; 95% CI 0.88–1.02, P = 0.15) shown in the pairwise meta-analysis of RIRS and PCNL for renal stones >2 cm reported by Zheng et al [73]. This is quite different from our meta-analysis results because Zheng and colleagues did not provide a clear quality assessment, there was a factor of publication bias, and it is presumed that the suitability of the effect model was not evaluated using the Labble plot. These conflicting results indicate that additional research is still needed.

Finally, without factoring the size and location of renal stones, the results presented in our study show that PCNL treatment resulted in the highest stone-free rate and SWL exhibited the lowest stone-free rate. Our study is unique in that three treatments were analyzed simultaneously using a network meta-analysis model. Furthermore, our study is judged to have great value because it is the first study to derive the superiority of a treatment using the rank test and because only studies with low bias and high quality were included in the analysis using quality assessment. Especially, in large stone (> 2 cm) and lower pole stone, PCNL can be superior to RIRS and SWL. EAU guidelines also recommended PCNL as the first-line treatment in large stone and lower pole stones. So far, the success rates of RIRS and SWL seem to not exceed that of PCNL. Based on our results, further research for treatments with higher stone-free rates will be necessary in the future.

The recently presented ECIRS is a treatment comprising a combination of PCNL and RIRS and is predicted to be capable of achieving a higher stone-free rate [74]. PCNL and RIRS should be the mainstay of interventional therapy for patients with renal stones. However, for some patients with bilateral disease, ECIRS may also be an effective treatment rather than bilateral PCNL or RIRS [75, 76]. Although PCNL is the most effect interventional therapy with the highest stone-free rate, careful patient selection is required because of the high invasiveness of this treatment. Indeed, recent reports highlight the advantage of reduced invasiveness in mini-PCNL and ultramini-PCNL treatments [77] and successful results in treatments with ECIRS performed with mini-PCNL [78]. In summary, PCNL is the most effective treatment, and RIRS is able to compensate for a lower stone-free rate than PCNL. For patients with a low stone-free rate in the recently presented nephrolithometry score [79], increasing the stone-free rate by using ECIRS should be the goal of interventional therapy in the future [76].

A limitation of our study is that no subgroup analysis was performed on the size and location of the renal stones. In the event that a subgroup analysis is performed, there is a possibility it may lead to different outcomes because the recommended treatments vary depending on the size and location of the renal stones. Some degree of publication bias was also a limitation of this study. However, Sutton et al. reviewed 48 articles from the Cochrane Database of Systematic Reviews and showed publication or related biases were common within the sample of meta-analyses assessed [80].

Another limitation is that the results reflected only the efficacy aspect of the stone-free rate and did not take into account the safety aspect of the treatments. Discriminating between merits and drawbacks of the treatment for a patient is clearly an important decision. Further studies that address these limitations are needed in the future.

## Conclusions

PCNL for renal stones resulted in the highest success and stone-free rate and ranked the highest of the treatments analyzed. In contrast, SWL ranked the lowest of the treatments because of its lowest success and stone-free rates. The complexity of individual patients considered in this meta-analysis may have played a role in the results. Future analyses should include patient selection criteria such as renal stone location.

## Supporting information

S1 Table PRISMA NMA checklist of Items to include when reporting a systematic review involving a network meta-analysis. (DOCX)

S2 Table Search strategy in PubMed. (DOCX)
