# Supplementary material for: Comparison of stone-free rates following shock wave lithotripsy, percutaneous nephrolithotomy, and retrograde intrarenal surgery for treatment of renal stones: A systematic review and network meta-analysis
Source: PLoS One. 2019 Feb 21;14(2):e0211316. doi: 10.1371/journal.pone.0211316 (PMC6383992; doi:10.1371/journal.pone.0211316)
Supplement: S2 Table — (DOCX) [file pone.0211316.s002.docx]

| S2 Table. Search startegy in PubMed | | |
| --- | --- | --- |
| Search | Query | Items found |
| #1 | Search **(extracorporeal shock wave lithotripsy) OR shock wave lithotripsy** | 12388 |
| #2 | Search **((percutaneous nephrolithotomy) OR nephrolithotomy) OR percutaneous** | 124958 |
| #3 | Search **((flexible ureteroscopy) OR flexible ureterorenoscopy) OR retrograde intrarenal surgery** | 1164 |
| #4 | Search **(renal stone) OR urolithiasis** | 3955 |
| #5 | Search **(rate) OR stone-free** | 1962180 |
| #6 | Search **((((#1) AND #2) AND #3) AND #4) AND #5** | 151 |
